# Post-COVID-19 era pathogen profiles and influencing factors for hospital patients with lower respiratory tract infections in Shenzhen, China

**DOI:** 10.3389/fcimb.2025.1703955

**Published:** 2025-12-05

**Authors:** Tong Li, Dandan Niu, Yingluan Zhang, Renli Zhang, Yongchao Guo, Tengyingzi Liu, Dana Huang, Zhen Zhang, Zhanhong Rao, Xiaolu Shi, Xiaomin Zhang, Tiejian Feng

**Affiliations:** 1 Shenzhen Center for Disease Control and Prevention, Shenzhen, China; 2 Shenzhen Uni-medical Technology Co., Ltd, Shenzhen, China; 3 Shenzhen Research Center for Communicable Disease Control and Prevention, Chinese Academy of Medical Sciences, Shenzhen, China; 4 Bao’an District Center for Disease Control and Prevention, Shenzhen, China

**Keywords:** lower respiratory tract infections, bronchoalveolar lavage fluid, etiology, the length of hospital stay, targeted next-generation sequencing

## Abstract

**Background:**

Lower respiratory tract infections (LRTIs) are associated with significant morbidity, hospitalizations, and mortality globally. The aim of this study was to investigate the causative pathogens and epidemiological characteristics of LRTIs including the factors influencing the severity of illness and the length of hospital stay (LOS).

**Methods:**

From November 2023 to June 2024, a prospective study including 121 patients was performed at four hospitals in Shenzhen, China. The bronchoalveolar lavage fluid (BALF) samples from inpatients with LRTIs were strictly collected and tested using targeted next-generation sequencing (tNGS). A total of 108 respiratory pathogens were included in the detection list.

**Results:**

Of the total inpatients, 89.3% tested positive for one or more pathogens (108/121). The three leading pathogens were *Staphylococcus aureus* (*S. aureus*), *Pseudomonas aeruginosa* (*P. aeruginosa*), and *Haemophilus influenzae* (*H. influenzae*). Patients aged over 70 years showed the highest pathogen detection rate (23/24, 95.8%), with *P. aeruginosa* being the most frequently detected pathogen in this age group (9/24, 37.5%). Profound sex differences were observed in *S. aeruginosa* infections. Multiple infections were found in 53.8% of all patients (65/121), in which 22.0% exhibited bacterial–viral co-infections (28/121). Positive interactions were identified in *P. aeruginosa*–*Acinetobacter baumannii or S. aureus*. High-risk factors of LOS were considerably associated with older age, multiple infections, pleural effusion, diabetes mellitus, hypertension, shortness of breath, and infection with *P. aeruginosa*, severe acute respiratory syndrome coronavirus 2 (SARS-CoV-2), or human herpes virus (HHV).

**Conclusions:**

Different age groups of hospitalized patients showed different pathogen profiles and types of co-infection, indicating the significance of age-targeted clinical medical practice. The most common pathogens that caused LRTIs were *S. aureus*, *P. aeruginosa*, and *H. influenzae*. Older age, multiple infections, pleural effusion, diabetes mellitus, hypertension, shortness of breath, and infection with *P. aeruginosa*, SARS-CoV-2, or HHV contribute to prolonged LOS. tNGS testing should be prioritized for immunocompromised and critically ill patients.

## Introduction

Lower respiratory tract infections (LRTIs), such as bronchiectasis, pneumonia, asthma, and chronic obstructive pulmonary disease (COPD), represent a major health concern worldwide, particularly in children and older adults, contributing significantly to morbidity, hospitalizations, and mortality (Woodhead, 2005). According to the World Health Organization (WHO)’s Global Health Estimates, LRTIs rank fourth among global causes of mortality with 2.6 million fatalities in 2019. In particular, the impact of LRTIs is more pronounced in low-income countries, where it ranks as the second-leading cause of death (World Health Organization, 2020). In mainland China, an annual average of 107,439 excess respiratory deaths occurred between 2010 and 2015, with the highest mortality in old adults (≥60 years) (Li et al., 2019). With the development of healthcare services and the public health system, the global mortality rate from LRTIs has decreased among children under 5 years old, but similar improvements have not been observed in adults over 70 years old (GBD, Lower, Respiratory et al., 2018). Therefore, minimizing the effect of LRTIs on human health continues to face great challenges (Oumei et al., 2018).

Conventional testing methods for LRTIs include direct microscopy, immunology, and molecular biology examination as well as pathogen isolation (Jianguo, 2021). However, there were 50.2%–67.1% of patients with no determined etiology (Musher et al., 2004; Shoar and Musher, 2020). Several factors contribute to diagnostic challenge: (1) the collection of low-quality samples; (2) limitations of conventional methods (poor sensitivity or low specificity); and (3) a narrow spectrum of pathogens tested. Because of the increased demand for highly sensitive and accurate diagnostic methods, next-generation sequencing has been the standard procedure in the clinical diagnosis of complicated infectious diseases (Yang et al., 2022). It has revolutionized the ability of clinical examination to rapidly detect a much wider diversity of pathogens (Church et al., 2020).

Nowadays, length of hospital stay (LOS) is a crucial indicator used for patient outcome assessment, which has multiple variables as effective factors, such as high age, male sex, aspiration pneumonia, temperature, humidity, and the different effects of antibiotics medicines in different age groups (Hossain et al., 2020; Agarwal et al., 2021; Wakabayashi et al., 2023). Long-term hospital stay may cause a large amount of medical burden and functional decline, and may significantly decrease quality of life (Khajehali and Alizadeh, 2017). Identifying the factors influencing LOS of patients with LRTIs may help doctors care for special groups and plan early personalized medical treatment (Giraldi et al., 2019).

Measures for COVID-19 control and the effect of COVID-19 on immune system might change the distribution of pathogen profiles, whereas the etiologic factors in hospitalized patients with LRTIs have not been sufficiently evaluated in the post-pandemic era. Targeted next-generation sequencing (tNGS) is a common sequencing method for the detection of pathogens in hospitals, which has the advantage of reducing human DNA interference and increasing sensitivity for detecting bacterial, virus, and fungi in human samples with an abundance of cells (Ee Auid-Orcid Hilt, 2022). This research aimed to investigate etiology and related determinants of LRTIs among hospitalized patients conducted from November 2023 to June 2024 in Shenzhen through a prospective cross-sectional study.

## Materials and methods

### Study design

A total of 142 hospitalized adult patients (age ≥ 18 years) with LRTIs were included in this prospective study over an 8-month period from November 2023 to June 2024 in four general hospitals in Shenzhen, China. The exclusion criteria were as follows: (1) non-infective pulmonary disease (*n* = 1); (2) incomplete data records or inadequate sample size (*n* = 19); and (3) treatment refusal (*n* = 1).

Bronchoalveolar lavage fluid (BALF) was collected by fiberoptic bronchoscopy within 48 h of admission from inpatients undergoing the clinically indicated procedure. For patients with multiple fiberoptic bronchoscopies, only the first sample was included. Then, the samples were transported under cold chain conditions within 72 h.

### Targeted next-generation sequencing

A total of 108 pathogens were detected using tNGS (59 bacteria, 39 viruses, and 10 fungi). Briefly, the tNGS workflow for high-throughput pathogen screening involved three general steps. Firstly, DNA and RNA were isolated from BALF followed by reverse transcription and library preparation. Multiplex polymerase chain reaction (PCR) was used for rapid microbial enrichment. Secondly, sequencing was performed on an Illumina MiniSeq platform using a rapid reagent kit and single-end sequencing. Finally, for generalizable and scalable use, a custom bioinformatic analysis pipeline was developed that automated data transfer and analysis (**Supplementary Material 1**).

### Covariables and definitions

Basic demographic information, including age and gender, and clinical data, including date of symptom onset, date of admission, date of discharge, and symptoms (cough, expectoration of sputum, fever, shortness of breath, rales, pleural effusion, hemoptysis, and chest pain), were obtained for the analysis via questionnaire (**Supplementary Material 2**). Medical history (diabetes mellitus, hypertension, etc.), the severity of illness, and LOS were supplied by the doctor. Age was stratified into three groups (18–49, 50–69, and ≥70 years).

### Ethics statement

This study was approved by the Ethics Committee of Shenzhen Center for Disease Control and Prevention. The design, conduct, and data collection of this study strictly adhered to the guidelines of the Ethics Committee and relevant international ethical guidelines. Informed consent was obtained from each participant or guardian.

### Data analysis

Statistical analysis was conducted using the Statistical Package for Social Science (SPSS, Release 26.0 standard version, IBM). A descriptive analysis was performed for demographic and clinical characteristics analysis, with results presented as interquartile range (IQR) for quantitative variables and percentages for categorical variables. Comparisons of qualitative variables were completed using the chi-square test or Fisher exact test. Multivariate associations between gender or pathogens and the type of illness were assessed by penalized logistic regression. Univariate associations between the severity of illness and potential covariates (age, gender, etc.) were assessed by penalized logistic regression; variables with *p* < 0.10 were subsequently entered into the multivariable model by penalized logistic regression. Univariate associations between LOS and potential covariates (age, gender, etc.) were assessed by Spearman’s rank correlation analysis or Mann–Whitney *U* test; variables with *p* < 0.10 were subsequently entered into the multivariable model by the Gamma generalized linear model.

## Results

### Participant characteristics and clinical diagnoses

A total of 121 hospitalized patients with LRTIs were included, their median age was 53 years (IQR, 37–66 years old), and the median LOS was 8 days (IQR, 5–11 days). Of these patients, 54.5% were male (66/121), 75.2% were diagnosed with pneumonia (91/121), and 16.5% were diagnosed with severe pneumonia (15/91) (Cao et al., 2018). The preponderant symptoms were cough (113/121, 93.4%), expectoration of sputum (92/121, 76.0%), fever (44/121, 36.4%), and shortness of breath (33/121, 27.3%) (**Table 1**).

**Table 1:** Demographic and clinical characteristics of inpatients diagnosed with low respiratory tract infections in Shenzhen, China.

Study population (%)	All	Positive samples	Pneumonia			Non-pneumonia		
			All	Monoinfection	Multiple infections	All	Monoinfection	Multiple infections
All	121	108 (89.3)	91 (75.2)	31 (34.1)	49 (53.8)	30 (24.8)	12 (40.0)	16 (53.3)
Age, years, median (IQR)								
18–49 years	50 (41.3)	42 (84.0)	42 (84.0)	22 (52.4)	14 (33.3)	8 (16.0)	4 (50.0)	2 (25.0)
50–69 years	47 (38.8)	43 (91.5)	31 (66.0)	6 (19.4)	21 (67.7)	16 (34.0)	6 (37.5)	10 (62.5)
≥70 years	24 (19.8)	23 (95.8)	18 (75.0)	3 (16.7)	14 (77.8)	6 (25.0)	2 (33.3)	4 (66.7)
Gender								
Male	66 (54.5)	61 (92.4)	50 (75.8)	18 (36.0)	28 (56.0)	16 (24.2)	7 (43.8)	8 (50.0)
Female	55 (45.5)	47 (85.5)	41 (74.5)	13 (31.7)	21 (51.2)	14 (25.5)	5 (35.7)	8 (57.1)
Symptom								
Cough	113 (93.4)	101 (89.4)	85 (75.2)	27 (31.8)	48 (56.5)	28 (24.8)	11 (39.3)	15 (53.6)
Expectoration of sputum	92 (76.0)	63 (68.5)	67 (72.8)	2 (3.0)	38 (56.7)	25 (27.2)	9 (36.0)	14 (56.0)
Fever (≥37.5°C)	44 (36.4)	39 (88.6)	39 (88.6)	10 (25.6)	24 (61.5)	5 (11.4)	1 (20.0)	4 (80.0)
Shortness of breath	33 (27.3)	30 (90.9)	23 (69.7)	7 (30.4)	14 (60.9)	10 (30.3)	2 (20.0)	7 (70.0)
Rales	21 (17.4)	21 (100.0)	15 (71.4)	5 (33.3)	10 (66.7)	6 (28.6)	1 (16.7)	5 (83.3)
Pleural effusion	19 (15.7)	18 (94.7)	17 (89.5)	5 (29.4)	12 (70.6)	2 (10.5)	1 (50)	0 (0.0)
Hemoptysis	8 (6.6)	7 (87.5)	4 (50.0)	1 (25.0)	2 (50.0)	4 (50.0)	3 (75)	1 (25)
Chest pain	6 (5.0)	4 (66.7)	5 (83.3)	1 (20.0)	3 (60.0)	1 (16.7)	0 (0.0)	0 (0.0)

### Pathogen positive rates

In total, 89.3% of 121 hospitalized patients with LRTIs were positive for one or more pathogens, with the highest positive rate in people aged ≥70 years (23/24, 95.8%), followed by 91.5% in adults aged 50–69 years (43/47), and 84.0% in those aged 18–49 years (42/50). There was no statistical difference among the positive rates in groups between age, gender (61/66, 92.4% in male *vs*. 47/55, 85.5% in female), or case type (80/91, 87.9% in pneumonia *vs*. 28/30, 93.3% in non-pneumonia) (*p* > 0.05). Single pathogen infection was detected in 35.5% of all patients (43/121), with the highest positivity rate observed in adults aged 18–49 years (22/42, 52.4% in pneumonia *vs*. 4/8, 50.0% in non-pneumonia). In contrast, 53.7% of all patients exhibited multiple pathogen infections (65/121), with the highest prevalence found in adults aged ≥70 years (**Table 1**).

### Pathogen profiles

**Figure 1:**
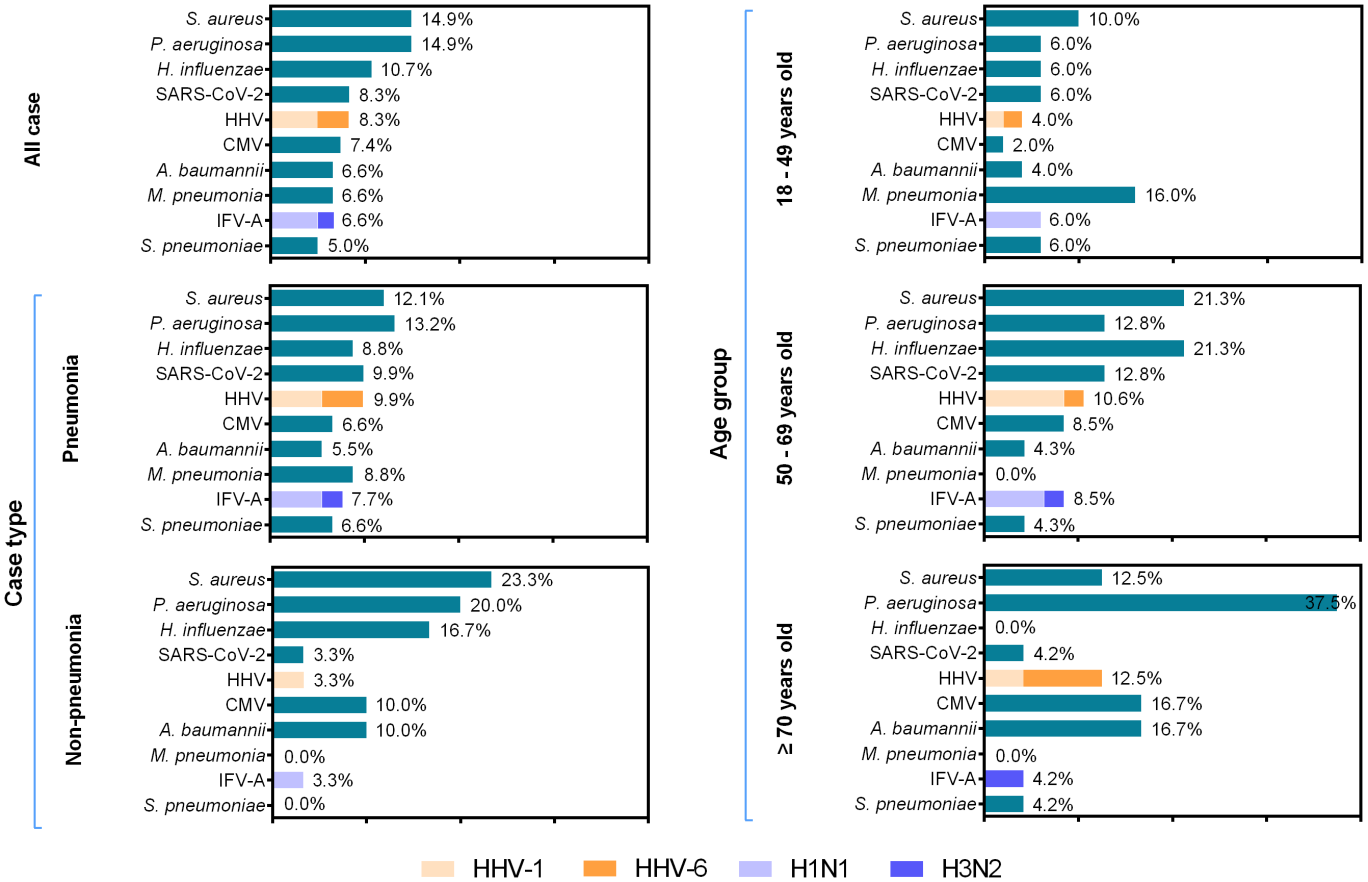
Pathogen distribution across different age groups among 121 inpatients with low respiratory tract infections, between November 2023 and June 2024. The length of colored bars and the number behind indicate the rates of each pathogen in different age groups. For IFV and HHV, the proportion of each subtype is indicated by the colored bar.

The most commonly detected pathogen among 121 hospitalized patients with LRTIs was *Staphylococcus aureus* (*S. aureus*) (18/121, 14.9%), followed by *Pseudomonas aeruginosa* (*P. aeruginosa*) (18/121, 14.9%), *Haemophilus influenzae* (*H. influenzae*) (13/121, 10.7%), severe acute respiratory syndrome coronavirus 2 (SARS-CoV-2) (10/121, 8.3%), human herpes virus (HHV) (10/121, 8.3%), cytomegalovirus (CMV) (9/121, 7.4%), *Acinetobacter baumannii* (*A. baumannii*) (8/121, 6.6%), *Mycoplasma pneumoniae* (*M. pneumoniae*) (8/121, 6.6%), influenza A virus (IFV-A) (8/121, 6.6%), and *Streptococcus pneumoniae* (*S. pneumoniae*) (6/121, 5.0%). In the HHV-positive group, the positive rates of HHV-1 and HHV-6 were 60.0% and 40.0%, respectively. Further genotyping analysis for IFV-A revealed that H1N1 was the predominant pathogen, accounting for 75.0% (6/8). *M. pneumoniae* was the predominant pathogen among adults aged 18–49 years, whereas *H. influenzae* exhibited the highest detection rate in the 50–69 year age group. *S. aureus* was the only pathogen with an infection rate exceeding 10% across all age groups, and demonstrated the highest infection prevalence in the cohort aged 50–69 years (10/47, 21.3%). Being positive for *P. aeruginosa* ranked first for people aged over 70 years old (9/24, 37.5%). The most frequently identified pathogen among inpatients with pneumonia was *P. aeruginosa* (12/91, 13.2%), followed by *S. aureus* (11/121, 12.1%), SARS-CoV-2 (9/121, 9.9%), and HHV (9/121, 9.9%). In the non-pneumonia inpatient group, the top-ranked pathogens were *S. aureus* (7/30, 23.3%), *P. aeruginosa* (6/30, 20.0%), and *H. influenzae* (5/30, 16.7%) (**Figure 1**).

**Figure 2:**
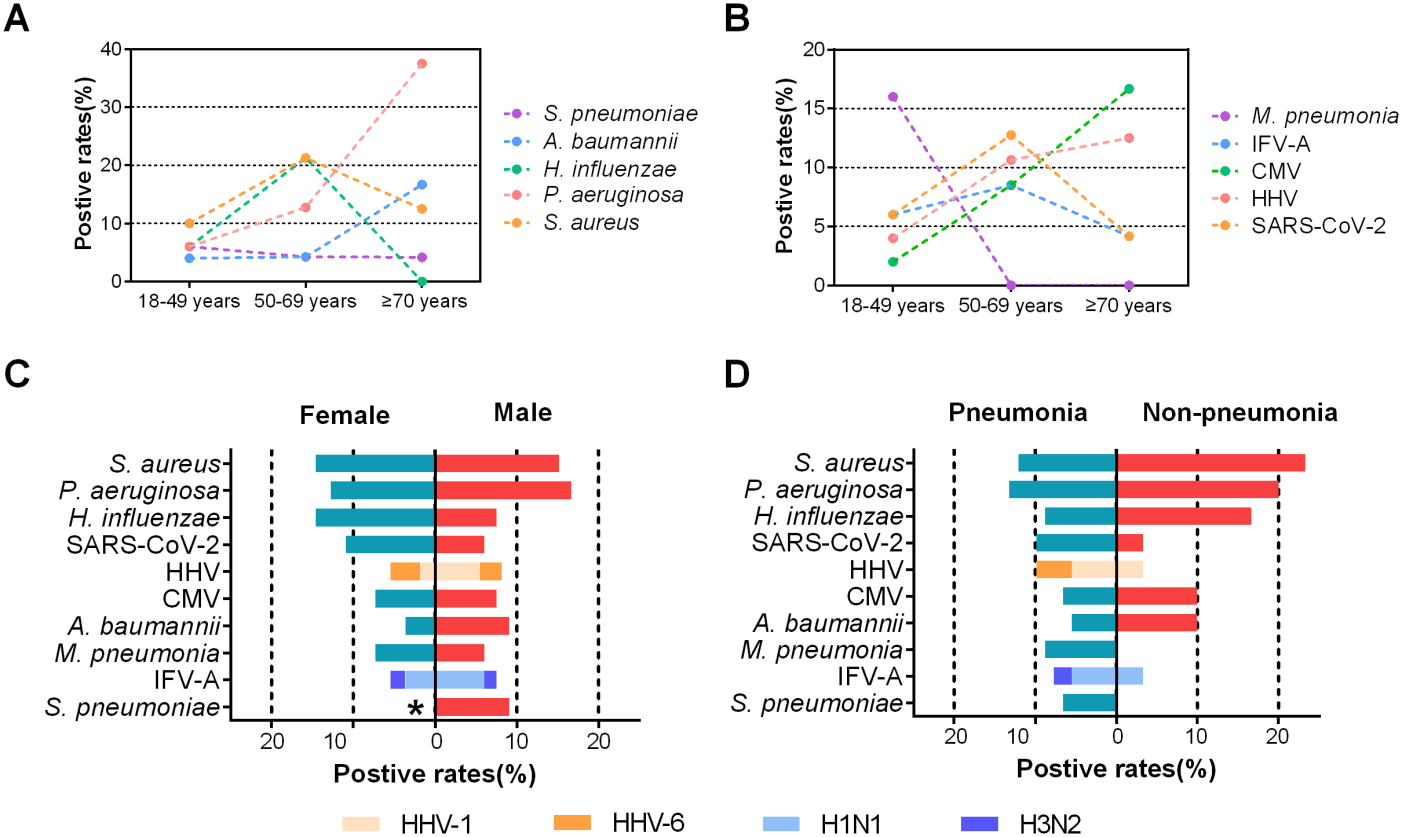
Comparison of the positive rates of pathogens with different age, case type, and gender groups in Shenzhen, China. **(A)** Prevalence of bacterial pathogens in different age groups. **(B)** Prevalence of viral and other pathogens in different age groups. **(C)** Comparison of the positive rates of pathogens in inpatients with male and female in Shenzhen, China. **(D)** Comparison of the positive rates of pathogens in inpatients with pneumonia and non-pneumonia in Shenzhen, China. The significant difference of the positive rate is indicated. For IFV and HHV, the proportion of each subtype is indicated by the colored bar. *p < 0.05.

tNGS analysis revealed an age-related rise in *P. aeruginosa* detection, whereas *M. pneumoniae* was exclusively detected in participants aged 18–49 years (*p* < 0.05). Moreover, *H. influenzae* exhibited high detection rates in individuals aged 50–69 years (*p* < 0.05) (**Figures 2A, B**). Among male patients, the pathogens with high detection rates included *P. aeruginosa* (11/66, 16.7%), *S. aureus* (10/66, 15.2%), *S. pneumoniae* (6/66, 9.1%), and *A. baumannii* (6/66, 9.1%). For female patients, the most frequently detected pathogens were *S. aureus* (8/55, 14.5%), *H. influenzae* (8/55, 14.5%), *P. aeruginosa* (7/55, 12.7%), and SARS-CoV-2 (6/55, 10.9%). Profound sex differences were observed in *S. pneumoniae* infection (9.1% in male *vs*. 0% in female, *p* < 0.05) (**Supplementary Material 3**). There were no significant differences in positive rates of pathogens between patients with pneumonia and those without (*p* > 0.05) (**Figures 2C, D**).

**Figure 3:**
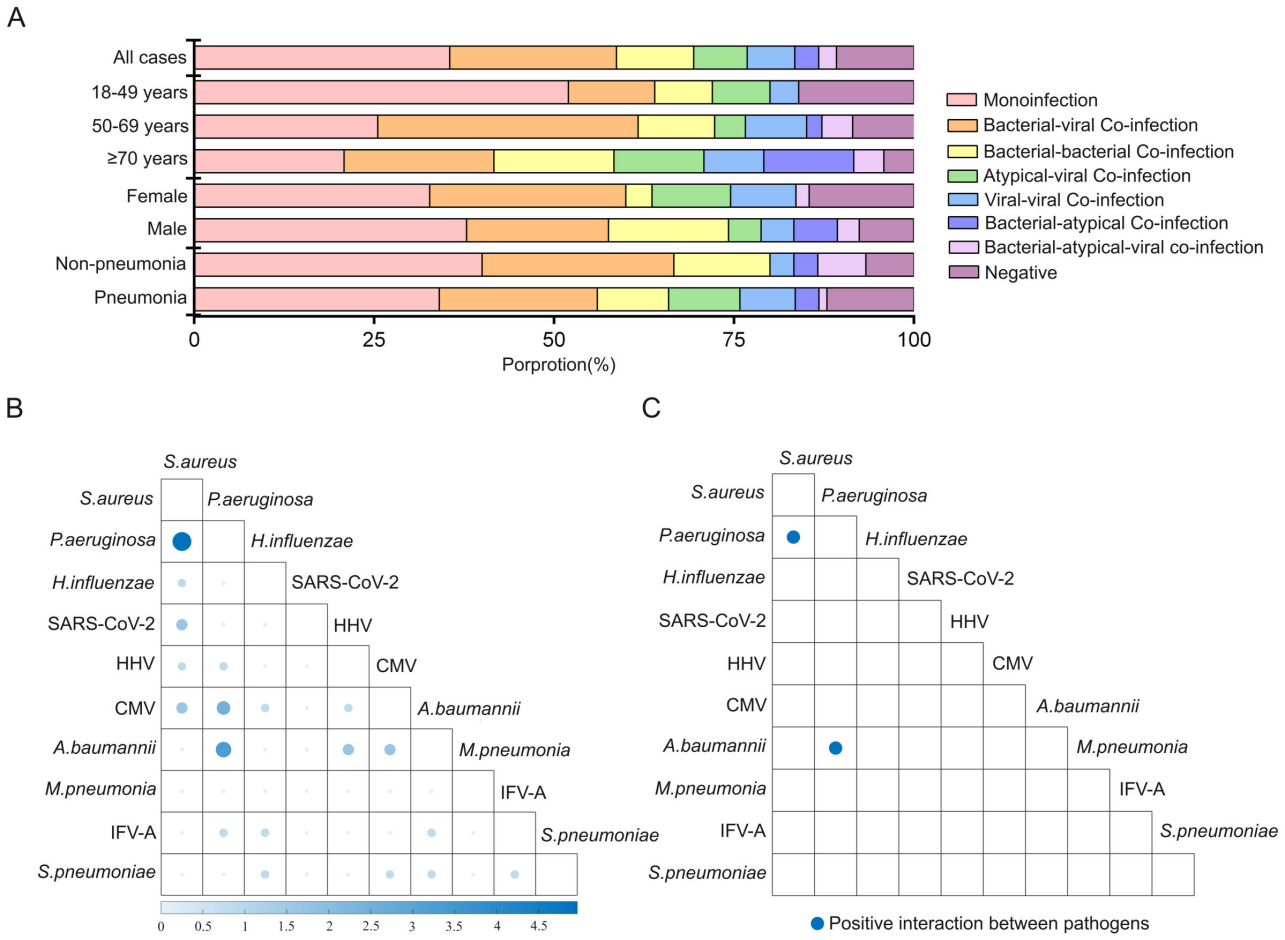
Prevalence of pathogens in monoinfection and co-infection in inpatients with low respiratory tract infections in Shenzhen, China. **(A)** Positive proportion of bacterial–bacterial co-infection, viral–viral co-infection, bacterial–viral co-infection, atypical–viral co-infection, bacterial–atypical co-infection, bacterial–atypical–viral co-infection, and monoinfection in different age, gender, and case type groups. **(B)** Heatmap of the co-infection rate of pathogens. Bigger size and darker color of the circles indicate higher co-infection rates between two pathogens. **(C)** The interactions among pathogens are estimated by host-scale logistic regressions. Positive interactions with two-sided *p* < 0.05 were denoted in blue color. The interaction was determined as significant both when without adjusting for multi-pathogens and when adjusting for multi-pathogens. The logistic analysis included the top 10 pathogens in the spectrum. “Atypical” refers to non-viral and non-bacterial pathogens, including *M. pneumoniae*, *C*. *psittaci*, Legionella, *P. jirovecii*, *A*. *fumigatus*, and *C*. *albicans*.

Multiple pathogen co-infections were found in 53.8% of all patients, in which 22.0% exhibited bacterial–viral co-infections, 9.9% showed bacterial–bacterial co-infections, 9.9% displayed atypical–viral co-infections, 7.7% showed viral–viral co-infections, 3.3% exhibited bacterial–atypical co-infections, and 1.1% showed bacterial–atypical–viral co-infections (**Figure 3A**). “Atypical” referred to *M. pneumoniae*, *C. psittaci*, Legionella, *P. jirovecii*, *A. fumigatus*, and *C. albicans*. Among these, bacterial–viral co-infections were the most common, particularly in the group aged 50–69 years. A higher monoinfection rate was observed in young adults compared to other age groups. There were no significant differences in co-infection rates between gender or case types (*p* > 0.05).

The most frequently observed co-infection involved *S. aureus* and *P. aeruginosa* (**Figure 3B**). The predominant co-infections were *S. aureus*–*P. aeruginosa*, *P. aeruginosa*–*A. baumannii*, and CMV–*P. aeruginosa*. Notably, positive interaction was identified in bacterial–bacterial co-infection (*S. aureus*–*P. aeruginosa* and *P. aeruginosa*–*A. baumannii*) (**Figure 3C**; **Supplementary Material 4**).

### Risk factors for the severity of pneumonia and the length of hospital stay

Multivariate analysis revealed that older age [odds ratio (OR) 1.050, 95% confidence interval (CI) 1.012–1.094], male (OR 4.188, 95% CI 1.100–23.021), shortness of breath (OR 14.971, 95% CI 3.487–108.717), and SARS-CoV-2 (OR 8.198, 95% CI 1.335–52.773) remained independently associated with the high risk of progressing to severe pneumonia (**Table 2**; **Supplementary Material 5**).

**Table 2:** Multivariate analysis of the severity of pneumonia.

Factor	Firth-OR	CI (95%)	*p*-value
Basic information			
Age	1.050	1.012 to 1.094	0.008
Gender	4.188	1.100 to 23.021	0.035
Diabetes mellitus	1.530	0.357 to 6.073	0.555
Hypertension	2.346	0.528 to 9.554	0.251
Infection type^a^			
Multiple infections	2.168	0.592 to 9.568	0.248
Symptom^a^			
Fever	3.164	0.795 to 14.634	0.103
Shortness of breath	14.971	3.487 to 108.717	<0.001
Pleural effusion	3.469	0.492 to 23.723	0.201
Pathogen type^a^			
SARS-CoV-2	8.198	1.335 to 52.773	0.024
HHV	4.424	0.825 to 22.759	0.808

OR, odds ratio.

a Adjusted for age, gender, diabetes mellitus and hypertension.

**Figure 4:**
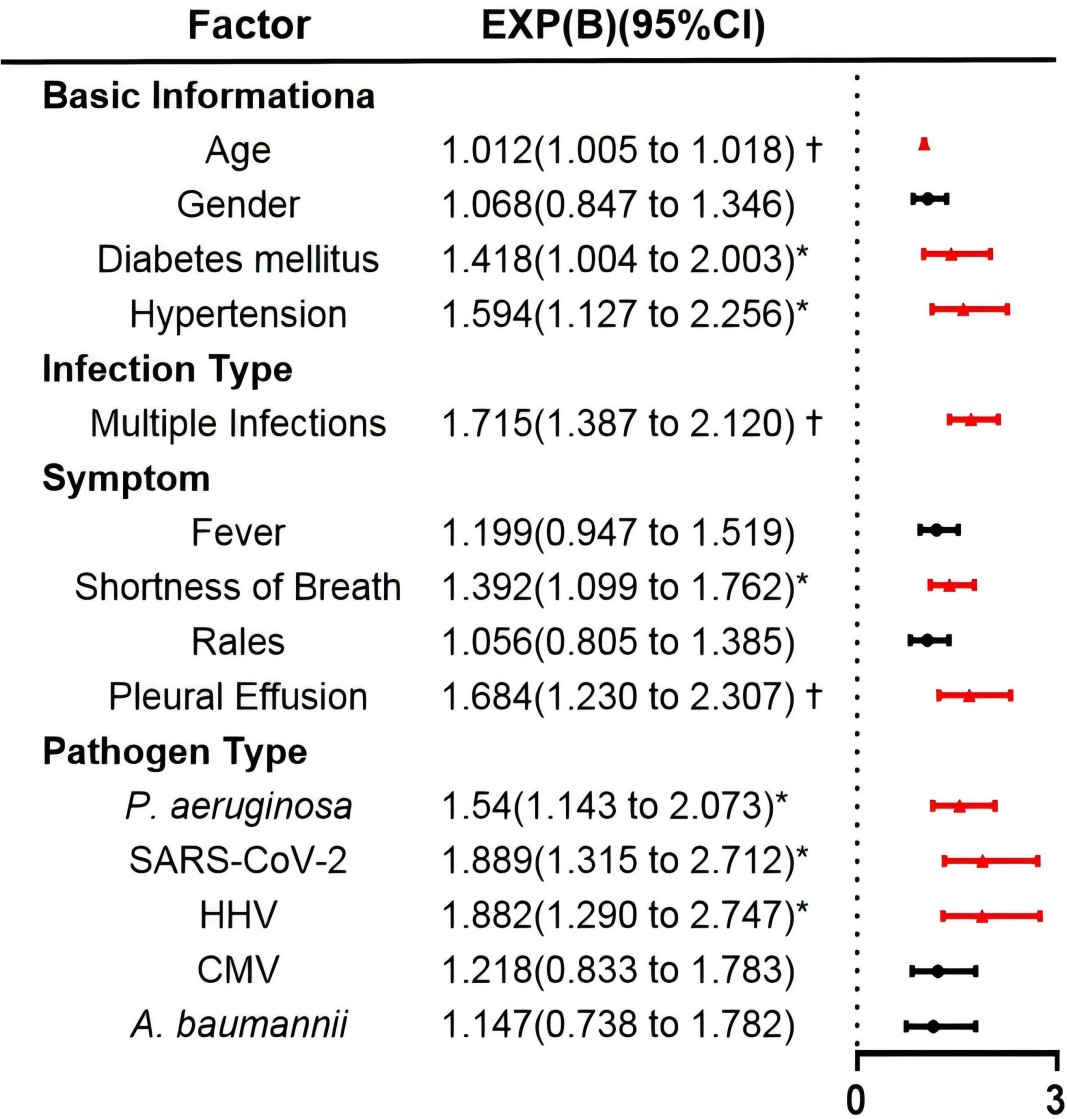
Forest plot summary of gamma generalized linear model of risk factors for length of hospital stay. *p < 0.05, †*p* < 0.001.

Multivariate analysis revealed that age (OR 1.012, 95% CI 1.005–1.018), multiple infections (OR 1.715, 95% CI 1.387–2.120), and pleural effusion (OR 1.684, 95% CI 1.230–2.307) were significantly associated with longer LOS (*p* < 0.001). The results indicated that diabetes mellitus (OR 1.418, 95% CI 1.004–2.003), hypertension (OR 1.594, 95% CI 1.127–2.256), shortness of breath (OR 1.392, 95% CI 1.099–1.762), *P. aeruginosa* (OR 1.540, 95% CI 1.143–2.073), SARS-CoV-2 (OR 1.889, 95% CI 1.315–2.712), and HHV (OR 1.882, 95% CI 1.290–2.747) were predictors of prolonged LOS (*p* < 0.05) (**Figure 4**; **Supplementary Material 6**).

## Discussion

This study systematically analyzed the pathogen characteristics and explored the risk factors associated with pneumonia severity and LOS among 121 inpatients with LRTIs in Shenzhen, China. The detection rate of pathogens in this study was 89.3%, close to that of 84.3%–92.7% reported in previous studies in China (Sun et al., 2024; Yin et al., 2024). In comparison with conventional technologies, such as PCR, culture, and antigen detection, tNGS showcases its advantages in detecting a wide array of both common and infrequent pathogens, including viruses, bacteria, and fungi, avoiding missed diagnosis and identifying mixed infection, providing a more accurate assessment of the infection landscape (Ee Auid-Orcid Hilt, 2022). Therefore, the improvement of detected technologies will help in accurately determining etiological diagnoses.

*S. aureus* and *P. aeruginosa*, the upper respiratory tract colonizers, were frequently detected in patients older than 50 years, which were increasingly recognized as important opportunistic pathogens and commonly associated with nosocomial infections (Chen et al., 2021). Their pathogenicity is influenced by specific resistance genes, biofilm formation, immune evasion, and toxin production, which contribute to their virulence to patients and tolerance to various antimicrobial agents (Gu et al., 2019). Patients with Pseudomonadales infection display an abundant microbiota with decreased richness and diversity, which may be difficult to treat (Xia et al., 2023). Therefore, drug resistance needs to be taken into account in the use of medicine. In line with early research from China, *H. influenzae* was the third-ranked pathogen in our population (Jianguo, 2021). In addition, *M. pneumoniae* was the major causative agent among young people, which was consistent with the trend in Poland (Rastawicki et al., 1998). In the univariate analysis, results showed that men are more susceptible to *S. pneumoniae* infection than women. In the age-adjusted analysis, a statistically significant association was observed between male gender and *S. pneumoniae* infection (OR 12.49, 95% CI 0.94–1,664.47, *p* = 0.019) (**Supplementary Material 3**). This might be attributed to lower IgG1, IgG2, and IgA1 levels in male patients in comparison to female patients (Knežević et al., 2022). However, the extraordinarily wide CI indicates substantial uncertainty in the magnitude of this effect, which is attributable to a quasi-complete separation in the data with no observed cases in the female subgroup.

It was found that the rates of bacterial–viral co-infection were more common. This age-dependent difference might reflect the different pathogen susceptibilities and complex pathogenic interactions. Viral infections could increase the risk and severity of bacterial infections by impairing mucociliary clearance, enhancing bacterial binding, upregulating host molecules, increasing bacteria adherence and internalization, compromising the epithelial barrier function, or causing immunological aberrances (Bellinghausen et al., 2016). On the other hand, bacterial infection could change the susceptibility and inflammatory response of the respiratory system to viruses by regulating the expression of viral receptors or altering virulence and structural modifications of viruses (Smith et al., 2014; Gulraiz et al., 2015). These interactions may be an underlying cause of the pathogenesis of bacterial–viral co-infection, leading to exacerbations of chronic disease and higher mortality rates (Cawcutt and Kalil, 2017).

Multiple infections significantly increased the severity of pneumonia and emerged as a key factor contributing to prolonged LOS. It was hypothesized that respiratory symptoms triggered by one pathogen might enhance the dispersal of another through aerosol generation and contribute to increased infectivity (Ferrari et al., 2024). The primary bacterial–bacterial synergistic effects were observed between *P. aeruginosa* and *A. baumannii* or *S. aureus*. *S. aureus* and *P. aeruginosa* were instead the most present in bacterial co-detections. Regrettably, it is hard to distinguish the sequential relationship of pathogen infection owing to the limited availability of BALF. The co-infection rate with *P. aeruginosa* and *S. aureus* was 83.3% in patients over 50 years old, which might be attributed to population susceptibility and opportunistic infection (Chen et al., 2021; Wei et al., 2023).

It has been found that inpatients with diabetes mellitus and hypertension had longer hospital stays, which is associated with a high risk of morbidity and mortality by respiratory infections. Hyperglycemia induces multiple pathophysiological alterations—including systemic inflammation, surfactant protein dysfunction, lung parenchymal damage, metabolic dysregulation, and immunosuppression—which collectively enhance host susceptibility to pathogens and accelerate viral replication post-infection (Wang et al., 2019).

Several critical limitations should be noted. Firstly, the sample size was small—a larger sample size with LRTIs might give a better understanding of the roles of these organisms. The external validity and statistical power of our findings are constrained by both sample-size limitations and model underperformance, particularly the statistical model’s modest explanatory capacity. Secondly, prior antimicrobial therapy of inpatients was 72.7%, which is similar to previous studies in Cameroon and China (Lanaspa et al., 2017; Giraldi et al., 2019). Empiric therapy and the use of broad-spectrum antibiotics might be biased for real pathogenesis and the pathogen frequencies reported. Thirdly, because of the high sensitivity and broad detectable pathogens of tNGS, both colonizing microorganisms and pathogenicity can be detected, making it challenging to distinguish the leading cause of the disease. Finally, BALF was collected within 48 h of admission, which might fail to capture the dynamic evolution and could not account for the potential impact of subsequent hospital-acquired infections on both clinical severity and LOS. Despite adjustments for known confounders, unmeasured or residual confounding (e.g., detailed immune status, timing of treatment initiation, and smoking) may influence both pathogen detection and outcomes, potentially accounting for the observed associations.

## Conclusion

In general, the three leading pathogens for LRTIs detected in BALF samples were *S. aureus*, *P. aeruginosa*, and *H. influenzae*. The infection pattern of respiratory pathogens was significantly variable in different age groups. The high detection rates of opportunistic pathogens in hospitalized patients were emphasized. Compared with other groups, patients with older age, male, shortness of breath, and infection with SARS-CoV-2 have significantly increased the likelihood of exacerbating pneumonia. The major factors contributing to prolong LOS included older age, multiple infections, pleural effusion, diabetes mellitus, hypertension, shortness of breath, and infection with *P. aeruginosa*, SARS-CoV-2 or HHV. This study provided a more comprehensive and novel epidemiology insight into the etiology of LRTIs for better clinical treatment in the post-pandemic era. Measurement of the background prevalence of normal controls might be helpful to clarify the causal relationships in future work. Meanwhile, given the limited availability of tNGS platforms in most hospitals and the higher per-sample cost compared with PCR, we recommend that future implementation strategies prioritize immunocompromised and critically ill patients for tNGS testing.

## Data Availability

Publicly available datasets were analyzed in this study. This data can be found here: https://bigd.big.ac.cn/gsa/browse/CRA021381.
