# Supplementary material for: Post-COVID-19 era pathogen profiles and influencing factors for hospital patients with lower respiratory tract infections in Shenzhen, China
Source: Front Cell Infect Microbiol. 2025 Dec 5;15:1703955. doi: 10.3389/fcimb.2025.1703955 (PMC12714876; doi:10.3389/fcimb.2025.1703955)
Supplement: Supplementary file 1 [file Table1.docx]

**Supplementary Material 1**

**Table S1** Pathogen Detection Panel (108 Pathogens)

| **Type** | **Pathogen** | |
| --- | --- | --- |
| **Bacteria(50)** | Streptococcus pneumoniae | Elizabethkingia meningoseptica |
|  | Streptococcus pyogenes | Streptococcus agalactiae |
|  | Pseudomonas aeruginosa | Enterococcus faecalis |
|  | Haemophilus influenzae | Enterococcus faecium |
|  | Enterobacter cloacae complex | Corynebacterium striatum |
|  | Escherichia coli | Staphylococcus epidermidis |
|  | Klebsiella Pneumoniae | Staphylococcus haemolyticus |
|  | Staphylococcus aureus | Mycobacterium tuberculosis complex |
|  | Rhodococcus equi | Mycobacterium avium |
|  | Moraxella catarrhalis | Mycobacterium intracellulare |
|  | Neisseria meningitidis | Mycobacterium gordonae |
|  | Nocardia | Mycobacterium kansasii |
|  | Nocardia farcinica | Mycobacterium scrofulaceum |
|  | Nocardia brasiliensis | Mycobacterium smegmatis |
|  | Nocardia concava | Mycobacterium chelonae |
|  | Nocardia cyriacigeorgica | Mycobacterium xenopi |
|  | Tropheryma whipplei | Mycobacterium abscessus |
|  | Klebsiella aerogenes | Mycobacterium fortuitum |
|  | Klebsiella oxytoca | Salmonella enterica subsp. Enterica |
|  | Bordetella pertussis | Stenotrophomonas maltophilia |
|  | Bordetella parapertussis | Burkholderia cepacia |
|  | Bordetella bronchiseptica | Burkholderia pseudomallei |
|  | Bordetella holmesii | Serratia marcescens |
|  | Legionella pneumophila | Francisella tularensis |
|  | Acinetobacter baumannii | Proteus mirabilis |
| **Virus(39)** | Human adenovirus B | Severe acute respiratory syndrome coronavirus |
|  | Human adenovirus C | Severe acute respiratory syndrome coronavirus 2 |
|  | Human adenovirus E | Influenza A virus |
|  | Human herpes virus 1 | Influenza B virus |
|  | Human herpes virus 2 | Influenza C virus |
|  | Varicella-zoster viru | Rubella virus |
|  | Epstein-Barr virus | Mumps virus |
|  | Cytomegalovirus | Measles virus |
|  | Human herpes virus 6 | Human parainfluenza virus type 1 |
|  | Human herpes virus 7 | Human parainfluenza virus type 2 |
|  | Bocavirus | Human parainfluenza virus type 3 |
|  | Human parvovirus B19 | Human parainfluenza virus type 4 |
|  | BK polyomavirus | Enterovirus |
|  | JC polyomavirus | Enterovirus type A71 |
|  | WU Polyomavirus | Enterovirus D68 |
|  | Human Coronavirus 229E | Rhinovirus |
|  | Human Coronavirus NL63 | Human metapneumovirus |
|  | Human Coronavirus HUK1 | human respiratory syncytial virus A |
|  | Human Coronavirus OC43 | human respiratory syncytial virus B |
|  | Middle east respiratory syndrome |  |
| **Special pathogens(9)** | Chlamydia psittaci | Rickettsia prowazekii |
|  | Mycoplasmal pneumonia | Rickettsia rickettsii |
|  | Chlamydia pneumoniae | Coxiella burnetii |
|  | Rickettsiatsu tsugamushi | Orientia tsutsugamushi |
|  | Chlamydia trachomatis |  |
| **Fungus(10)** | Cryptococcus neoformans complex | Talaromyces mameffei |
|  | Cryptococcus gattii complex | Scedosporium apiospermum |
|  | Pneumocystis jirovecii | Candida albicans |
|  | Aspergillus flavus | Mucor racemosus |
|  | Aspergillus fumigatus | Rhizopus oryzae |

**Targeted next-generation sequencing (tNGS) workflow and bioinformatics analysis**

**1 Sample processing and nucleic acid extraction**

The tNGS was conducted using a clinically validated multiplex respiratory pathogen panel (Shenzhen Uni-medica Technology Co., Ltd, China) covering 59 bacteria, 39 viruses, and 10 fungi. (1) 1 mL of specimen was centrifuged at 10,000 rpm for 5 min, and approximately 700 μL of supernatant was discarded.

(2) The residual sample was transferred to a lysis tube containing 300 μL of lysis buffer and 30 μL of dithiothreitol and then homogenized by a tissue grinder homogenizer (TGrinder H24, TIANGEN, China) under standard oscillation cycles (6 M/S oscillation for 30 sec, interval 30 sec, 3 cycles).

(4) Aspirate 550 μL of supernatant for automated nucleic acid extraction.​

(5) Co-extraction of DNA and RNA was carried out using an automated nucleic acid extractor (NFAST 96, Shenzhen Uni-medica Technology Co., Ltd, China). All extraction procedures included an internal positive control and a no-template water control to monitor potential contamination and extraction efficiency.

**2 Reverse transcription and target region amplification**

(1) Add 1 μL Random Hexamers, 2 μL 10× RT Mix, 2 μL RT Enzyme Mix and 15 μL template into PCR tubes.The reaction was carried out under the following conditions: 25°C for 2 min, 37°C for 5 min, 50°C for 15 min, and 85°C for 5 s.

(2) Add 1 μL BC 01-200, 8 μL PCR Mix, 6 μL PCR Enzyme Mix and 15 μL cDNA/DNA template into PCR tubes. The amplification protocol consisted of an initial denaturation at 95°C for 3 min, followed by 20 cycles of 95°C for 30 s, 60°C for 30 s, 57°C for 30 s, and 72°C for 30 s, with a final extension at 72°C for 3 min.

**3 Purification and library amplification**

(1) PCR products were purified using magnetic bead-based purification following the standard two-step binding and washing procedure with freshly prepared 80% ethanol. DNA was eluted in 20 μL of elution buffer.

(2) For library amplification, purified PCR products were mixed with universal primer (N501–N508) and UPM PCR buffer. The amplification program was as follows: 95°C for 3 min; 10 cycles of 98°C for 20 s, 60°C for 15 s, and 72°C for 30 s; and a final extension at 72°C for 3 min.

(3) A second round of magnetic bead purification was performed to remove primer dimers and small fragments. The final libraries were eluted in 18 μL of elution buffer.

**4 Quality control and sequencing**

(1) Measure 2 μL purified product using Qubit 3.0/4.0. Acceptance Criteria: ≥0.265 ng/μL.

(2) Determine the library by Agilent 2100 Bioanalyzer analysis using the High Sensitivity DNA Kit. The main peak should be between 500-600 bp.

(3) After passing quality control, the library is diluted and denatured, followed by sequencing on the Illumina MiniSeq platform using a single-end 100 bp read length mode.

(4)For contamination control, each experimental run includes a parallel blank (water) control. A pathogen is reported in the clinical sample only when its normalized read count is ≥10-fold higher than that detected in the corresponding blank control.

**5 Bioinformatics analysis pipeline**

A customized bioinformatics pipeline was developed for automated analysis and interpretation of sequencing data.

(1) Raw reads generated from the sequencer were first uploaded to a secure analysis server for data preprocessing, including adapter trimming , low-quality reads filterring, and low-complexity reads and redundancy removal.

(2)The resulting high-quality reads were then aligned against a primer reference database to identify and eliminate primer dimers and non-specific sequences. Subsequently, the filtered reads were mapped to an in-house validated pathogen sequence database for taxonomic classification, in which sequences were categorized as pathogen-specific or unclassified reads. The reporting threshold for a pathogen is set at≥20 normalized reads.

(3) QC: Q20≥90%, Q30≥85%, high-quality reads≥4000, and internal control/external control reads≥0.

(4) Once QC is passed, the results report can be automatically generated.
