# Supplementary material for: Post-COVID-19 era pathogen profiles and influencing factors for hospital patients with lower respiratory tract infections in Shenzhen, China
Source: Front Cell Infect Microbiol. 2025 Dec 5;15:1703955. doi: 10.3389/fcimb.2025.1703955 (PMC12714876; doi:10.3389/fcimb.2025.1703955)
Supplement: Supplementary file 2 [file Table2.docx]

**Supplementary Material 2**

**Table S2 Inspection Form**

**Submitting Organization：** **Sample Number：**

| Department | □Emergency □Infectious Diseases Department □ICU  □Hematology Department □Fever Clinic □Respiratory Department □Department of Pediatrics □GI Clinic □Gastroenterology Department  □Neonatology Department □Diarrhea Clinic □Neurology Department □Oncology Department □Other |
| --- | --- |
| **Personal Information**  Name Gender □Man □Female Date of Birth  Date of Onset Date of Consultation  Date of Admission Hospitalization Number  Clinic Number Preliminary Diagnosis  Temperature： ℃ ( □Axillary Temp □Forehead Temp □Ear Temp ）  Respiratory Symptoms： □Cough □Shortness of Breath □Sputum □Rhinorrhea □Other  Gastrointestinal Symptoms： □Vomiting □Diarrhea □Abdominal Pain □Nausea □Other  Neurological Symptoms： □Headache □Somnolence □Coma □Altered Mental Status □Other  Skin and Mucous Membrane Symptoms： □Jaundice □Rash □Bleeding □Other  Antibiotic Use： □No □Yes | |
| **Sample Type:** □Oral Swab □Nasopharyngeal Swab □Sputum □BALF □Other | |

Sample Collector (Doctor/Nurse): Collection Date：

Receiver at Lab (Lab Department): Receipt Date：
