# Supplementary material for: Post-COVID-19 era pathogen profiles and influencing factors for hospital patients with lower respiratory tract infections in Shenzhen, China
Source: Front Cell Infect Microbiol. 2025 Dec 5;15:1703955. doi: 10.3389/fcimb.2025.1703955 (PMC12714876; doi:10.3389/fcimb.2025.1703955)
Supplement: Supplementary file 3 [file Table3.docx]

**Supplementary Material 3**

**Table S2** ‌Univariate Analysis‌ of Pathogen Positive Rates between Male and Female Inpatients

| **Pathogen Type** | **Positive Rates(n, %)** | | **χ 2** | ***P* -value** | **Minimum Expected Count** |
| --- | --- | --- | --- | --- | --- |
|  | **Male**  **(N=66)** | **Female**  **(N=55)** |  |  |  |
| ***S. aureus*^a^** | 10(15.15) | 8(14.55) | 0.009 | 0.926 | 8.18 |
| ***P. aeruginosa*^a^** | 11(16.67) | 7(12.72) | 0.368 | 0.544 | 8.18 |
| ***H. influenzae^a^*** | 5(7.58) | 8(14.55) | 1.520 | 0.218 | 5.91 |
| **SARS-CoV-2^b^** | 4(6.06) | 6(10.91) | 0.401 | 0.527 | 4.55 |
| **HHV^b^** | 5(7.58) | 3(5.45) | 0.010 | 0.920 | 3.64 |
| **CMV^b^** | 5(7.58) | 4(7.28) | ＜0.001 | 1.000 | 4.09 |
| ***A. baumannii*^b^** | 6(9.09) | 2(3.34) | 0.697 | 0.404 | 3.64 |
| ***M. pneumoni*a^b^** | 4(6.06) | 4(7.28) | ＜0.001 | 1.000 | 3.64 |
| **IFV-A^b^** | 5(7.58) | 3(5.45) | 0.481 | 0.488 | 4.55 |
| ***S. pneumoniae*^b^** | 6(9.09) | 0(0.00%) | 3.509 | 0.061 | 2.73 |

^a^Analyzed by Pearson Chi-square test.

^b^Analyzed by Continuity Correction.

**Table S3** Multivariate Analysis‌ of Pathogen Positive Rates between Male and Female Inpatients(Adjusted for Age)

| **Pathogen Type** | **Firth-OR** | **CI(95%)** | ***P* -value** |
| --- | --- | --- | --- |
| ***S. aureus*** | 1.004 | 0.372 to 2.765 | *0.994* |
| ***P. aeruginosa*** | 1.092 | 0.387 to 3.190 | 0.868 |
| ***H. influenzae*** | 0.471 | 0.140 to 1.46 | 0.193 |
| **SARS-CoV-2** | 0.534 | 0.139 to 1.877 | 0.327 |
| **HHV** | 1.645 | 0.452 to 7.146 | 0.457 |
| **CMV** | 0.863 | 0.225 to 3.434 | 0.828 |
| ***A. baumannii*** | 1.976 | 0.469 to11.236 | 0.364 |
| ***M. pneumonia*** | 1.256 | 0.269 to 6.030 | 0.768 |
| **IFV-A** | 1.369 | 0.344 to 6.198 | 0.657 |
| ***S. pneumoniae*** | 12.463 | 1.405 to 1648.468 | 0.019 |

OR: Odds Ratio; CI: Confidence Intervals.


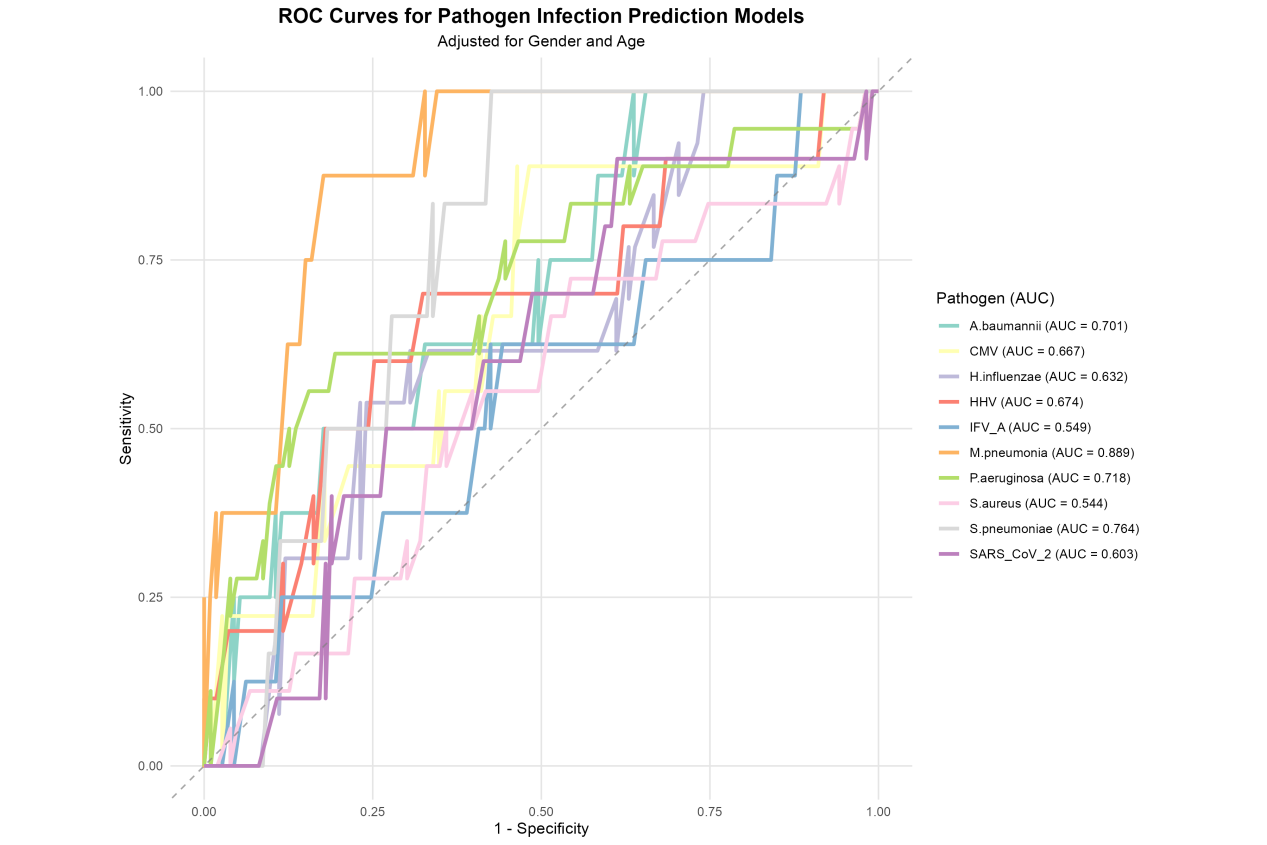


**Figure S1** ROC Curves for Multivariate Analysis‌ of Pathogen Positive Rates between Male and Female Inpatients(Adjusted for Age)

**Table S4** ‌Univariate Analysis‌ of Pathogen Positive Rates between Pneumonia and Non-pneumonia Inpatients

| **Pathogen Type^a^** | **Positive Rates(n, %)** | | **χ 2** | ***P* -value** | **Minimum Expected Count** |
| --- | --- | --- | --- | --- | --- |
|  | **Pneumonia**  **(N=91)** | **Non-**  **pneumonia**  **(N=30)** |  |  |  |
| ***S. aureus*** | 11(12.08) | 7(23.33) | 1.453 | 0.228 | 4.46 |
| ***P. aeruginosa*** | 12(13.18) | 6(20.00) | 0.337 | 0.539 | 4.46 |
| ***H. influenzae*** | 8(8.79) | 5(16.66) | 0.754 | 0.358 | 3.22 |
| **SARS-CoV-2** | 9(9.89) | 1(3.33) | 0.561 | 0.454 | 2.48 |
| **HHV** | 9(9.89) | 1(3.33) | 0.561 | 0.454 | 2.48 |
| **CMV** | 6(6.59) | 3(10.00) | 0.046 | 0.829 | 2.23 |
| ***A. baumannii*** | 5(5.49) | 3(10.00) | 0.192 | 0.662 | 1.98 |
| ***M. pneumoni*a** | 8(8.79) | 0(0.00) | 1.580 | 0.209 | 1.98 |
| **IFV-A** | 7(7.69) | 1(3.33) | 0.168 | 0.682 | 1.98 |
| ***S. pneumoniae*** | 6(6.59) | 0(0.00) | 0.917 | 0.338 | 1.49 |

^a^Analyzed by Continuity Correction.

**Table S5** Multivariate Analysis‌ of Pathogen Positive Rates between Pneumonia and Non-pneumonia Inpatients(Adjusted for Age and Gender)

| **Pathogen Type** | **Firth-OR** | **CI(95%)** | ***P* -value** |
| --- | --- | --- | --- |
| ***S. aureus*** | 0.460 | 0.167 to 1.324 | *0.145* |
| ***P. aeruginosa*** | 0.646 | 0.222 to 2.024 | 0.439 |
| ***H. influenzae*** | 0.495 | 0.156 to 1.678 | 0.247 |
| **SARS-CoV-2** | 2.340 | 0.505 to 22.501 | 0.305 |
| **HHV** | 2.507 | 0.529 to 24.448 | 0.271 |
| **CMV** | 0.655 | 0.171 to 2.948 | 0.557 |
| ***A. baumannii*** | 0.534 | 0.130 to 2.485 | 0.401 |
| ***M. pneumonia*** | 5.872 | 0.631 to 783.659 | 0.138 |
| **IFV-A** | 1.717 | 0.354 to 16.721 | 0.534 |
| ***S. pneumoniae*** | 4.685 | 0.504 to 623.612 | 0.207 |

OR: Odds Ratio; CI: Confidence Intervals.


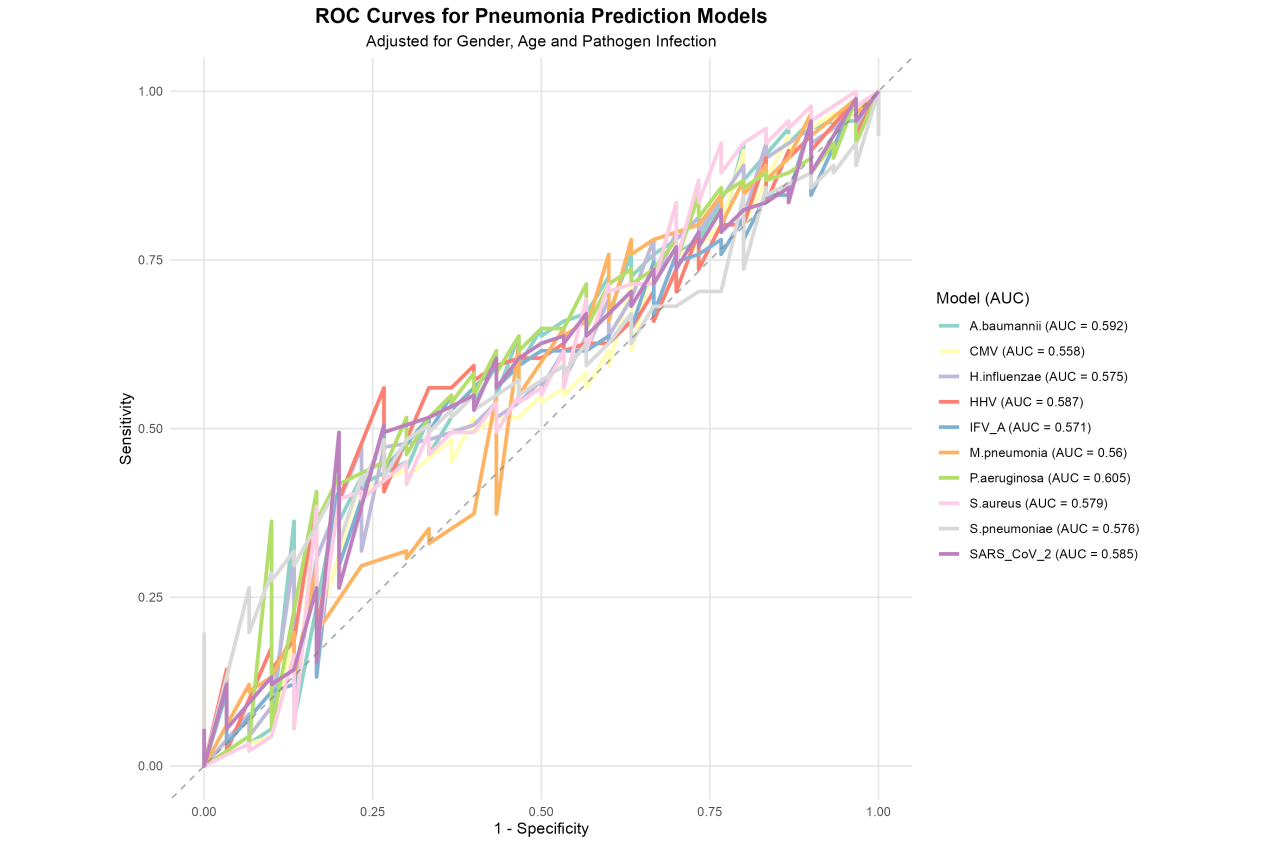


**Figure S2** ROC Curves for Multivariate Analysis‌ of Pathogen Positive Rates between Pneumonia and Non-pneumonia Inpatients(Adjusted for Age and Gender)
