# Supplementary material for: Post-COVID-19 era pathogen profiles and influencing factors for hospital patients with lower respiratory tract infections in Shenzhen, China
Source: Front Cell Infect Microbiol. 2025 Dec 5;15:1703955. doi: 10.3389/fcimb.2025.1703955 (PMC12714876; doi:10.3389/fcimb.2025.1703955)
Supplement: Supplementary file 4 [file Table4.docx]

**Supplementary Material 4**

To examine within-host co-infection patterns among the ten most prevalent pathogens, we applied a three-step logistic modelling strategy designed to minimise selection bias.(1) pairwise logistic regressions. For each target pathogen Y, we regressed Y on every non-Y pathogen X, adjusting for age, gender and medical history, yielding crude inter-pathogen associations.(2) full-pathogen logistic regressions. Each pathogen Y was modelled against all remaining pathogens simultaneously, again adjusting for age, gender and medical history, to account for mutual confounding.(3) consistency check. Associations that reached statistical significance (P < 0.05) in both steps were considered robust indicators of pathogen–pathogen interaction.

**Table S6** Univariate Logistic Regression Analysis Yielded Unadjusted Interactions for Other Pathogens.

| **Firth-OR** | ***S. aureus*** | ***P. aeruginosa*** | ***H. influenzae*** | **SARS-CoV-2** | **HHV** | **CMV** | ***A. baumannii*** | ***M. pneumonia*** | **IFV-A** | ***S. pneumoniae*** |
| --- | --- | --- | --- | --- | --- | --- | --- | --- | --- | --- |
| ***S. aureus*** |  |  |  |  |  |  |  |  |  |  |
| ***P. aeruginosa*** | 3.734^*^ |  |  |  |  |  |  |  |  |  |
| ***H. influenzae*** | 0.598 | 0.191 |  |  |  |  |  |  |  |  |
| **SARS-CoV-2** | 1.68 | 0.264 | 0.264 |  |  |  |  |  |  |  |
| **HHV** | 0.792 | 0.418 | 0.347 | 0.458 |  |  |  |  |  |  |
| **CMV** | 1.716 | 2.412 | 1.092 | 0.417 | 1.747 |  |  |  |  |  |
| ***A. baumannii*** | 0.237 | 9.537^*^ | 0.277 | 0.344 | 5.174 | 3.474 |  |  |  |  |
| ***M. pneumonia*** | 0.306 | 0.914 | 0.476 | 0.553 | 0.716 | 1.288 | 1.277 |  |  |  |
| **IFV-A** | 0.31 | 1.122 | 1.772 | 0.591 | 1.193 | 0.825 | 6.533 | 0.605 |  |  |
| ***S. pneumoniae*** | 0.36 | 0.34 | 3.112 | 0.836 | 0.388 | 3.233 | 2.814 | 0.769 | 3.933 |  |

OR: Odds Ratio

^*^*P*＜0.05

**Table S7** Multivariate Logistic Regression Analysis Yielded Adjusted Interactions for Other Pathogens.

| **Firth-OR** | ***S. aureus*** | ***P. aeruginosa*** | ***H. influenzae*** | **SARS-CoV-2** | **HHV** | **CMV** | ***A. baumannii*** | ***M. pneumonia*** | **IFV-A** | ***S. pneumoniae*** |
| --- | --- | --- | --- | --- | --- | --- | --- | --- | --- | --- |
| ***S. aureus*** | - | 4.643^*^ | 0.67 | 1.548 | 1.062 | 1.578 | 0.166 | 0.453 | 0.38 | 0.536 |
| ***P. aeruginosa*** | 4.656^*^ | - | 0.205 | 0.208 | 0.294 | 1.491 | 161.408^*^ | 0.431 | 0.756 | 0.437 |
| ***H. influenzae*** | 0.689 | 0.268 | - | 0.2 | 0.342 | 1.259 | 0.831 | 0.726 | 1.181 | 1.8 |
| **SARS-CoV-2** | 1.384 | 0.201 | 0.17 | - | 0.329 | 0.563 | 1.18 | 0.434 | 0.589 | 1.004 |
| **HHV** | 0.872 | 0.258 | 0.245 | 0.332 | - | 1.449 | 19.04 | 0.535 | 0.263 | 0.529 |
| **CMV** | 1.739 | 1.592 | 1.648 | 0.584 | 1.3 | - | 7.033 | 0.871 | 0.506 | 1.868 |
| ***A. baumannii*** | 0.091 | 13.706^*^ | 0.556 | 1.324 | 5.028 | 2.713 | - | 3.406 | 3.551 | 2.044 |
| ***M. pneumonia*** | 0.252 | 1.063 | 0.363 | 0.382 | 0.736 | 1.12 | 11.327 | - | 0.515 | 0.565 |
| **IFV-A** | 0.257 | 1.331 | 1.139 | 0.569 | 0.392 | 0.571 | 50.895 | 0.411 | - | 2.297 |
| ***S. pneumoniae*** | 0.412 | 0.24 | 1.636 | 0.885 | 0.61 | 3.5 | 4.86 | 0.514 | 1.891 | - |

OR: Odds Ratio

^*^*P*＜0.05
