# Supplementary material for: Post-COVID-19 era pathogen profiles and influencing factors for hospital patients with lower respiratory tract infections in Shenzhen, China
Source: Front Cell Infect Microbiol. 2025 Dec 5;15:1703955. doi: 10.3389/fcimb.2025.1703955 (PMC12714876; doi:10.3389/fcimb.2025.1703955)
Supplement: Supplementary file 5 [file Table5.docx]

**Supplementary Material 5**

**Table S8** ‌Univariate Analysis‌ of the Severity of Pneumonia

OR: Odds Ratio

| **Factor** | **Firth-OR** | **CI(95%)** | ***P* -value** |
| --- | --- | --- | --- |
| **Basic Information** |  |  |  |
| Age | 1.059 | 1.024 to 1.101 | ＜0.001 |
| Gender | 5.400 | 1.537 to 28.482 | 0.007 |
| Diabetes Mellitus | 3.960 | 1.145 to 12.821 | 0.031 |
| Hypertension | 3.250 | 0.857 to 10.981 | 0.080 |
| **Infection Type** |  |  |  |
| Multiple Infections | 3.707 | 1.167 to 15.111 | 0.025 |
| **Symptom** |  |  |  |
| Cough | 2.675 | 0.305 to 352.295 | 0.446 |
| Expectoration of Sputum | 1.868 | 0.521 to 9.955 | 0.361 |
| Fever | 2.944 | 1.016 to 9.038 | 0.047 |
| Shortness of Breath | 14.203 | 4.329 to 59.447 | ＜0.001 |
| Rales | 2.001 | 0.546 to 6.387 | 0.276 |
| Pleural Effusion | 3.342 | 0.980 to 10.588 | 0.054 |
| Heamoptysis | 0.374 | 0.003 to 3.276 | 0.446 |
| Chest Pain | 1.909 | 0.186 to 10.574 | 0.525 |
| **Pathogen Type** |  |  |  |
| *S. aureus* | 1.653 | 0.389 to 5.648 | 0.464 |
| *P. aeruginosa* | 1.653 | 0.389 to 5.648 | 0.464 |
| *H. influenzae* | 0.223 | 0.002 to 1.848 | 0.202 |
| SARS-CoV-2 | 3.715 | 0.817 to 14.462 | 0.085 |
| HHV | 6.050 | 1.485 to 23.328 | 0.014 |
| CMV | 2.457 | 0.425 to 10.465 | 0.282 |
| *A. baumannii* | 2.863 | 0.488 to 12.664 | 0.218 |
| *M. pneumonia* | 0.374 | 0.003 to 3.276 | 0.446 |
| IFV-A | 2.863 | 0.488 to 12.664 | 0.218 |
| *S. pneumoniae* | 4.218 | 0.682 to 21.17 | 0.112 |

**Table S9** Multivariate Analysis‌ of the Severity of Pneumonia

| **Factor** | **Firth-OR** | **CI(95%)** | ***P* -value** | **VIF** |
| --- | --- | --- | --- | --- |
| **Basic Information** |  |  |  |  |
| Age | 1.050 | 1.012 to 1.094 | 0.008 | 1.183 |
| Gender | 4.188 | 1.100 to 23.021 | 0.035 | 1.097 |
| Diabetes Mellitus | 1.530 | 0.357 to 6.073 | 0.555 | 1.226 |
| Hypertension | 2.346 | 0.528 to 9.554 | 0.251 | 1.077 |
| **Infection Type**^a^ |  |  |  |  |
| Multiple Infections | 2.168 | 0.592 to 9.568 | 0.248 | 1.145 |
| **Symptom**^a^ |  |  |  |  |
| Fever | 3.164 | 0.795 to 14.634 | 0.103 | 1.163 |
| Shortness of Breath | 14.971 | 3.487 to 108.717 | ＜0.001 | 1.121 |
| Pleural Effusion | 3.469 | 0.492 to 23.723 | 0.201 | 1.242 |
| **Pathogen Type**^a^ |  |  |  |  |
| SARS-CoV-2 | 8.198 | 1.335 to 52.773 | 0.024 | 1.032 |
| HHV | 4.424 | 0.825 to 22.759 | 0.808 | 1.057 |

OR: Odds Ratio;VIF: Variance Inflation Factor

^a^Adjusted for age, gender, diabetes mellitus and hypertension


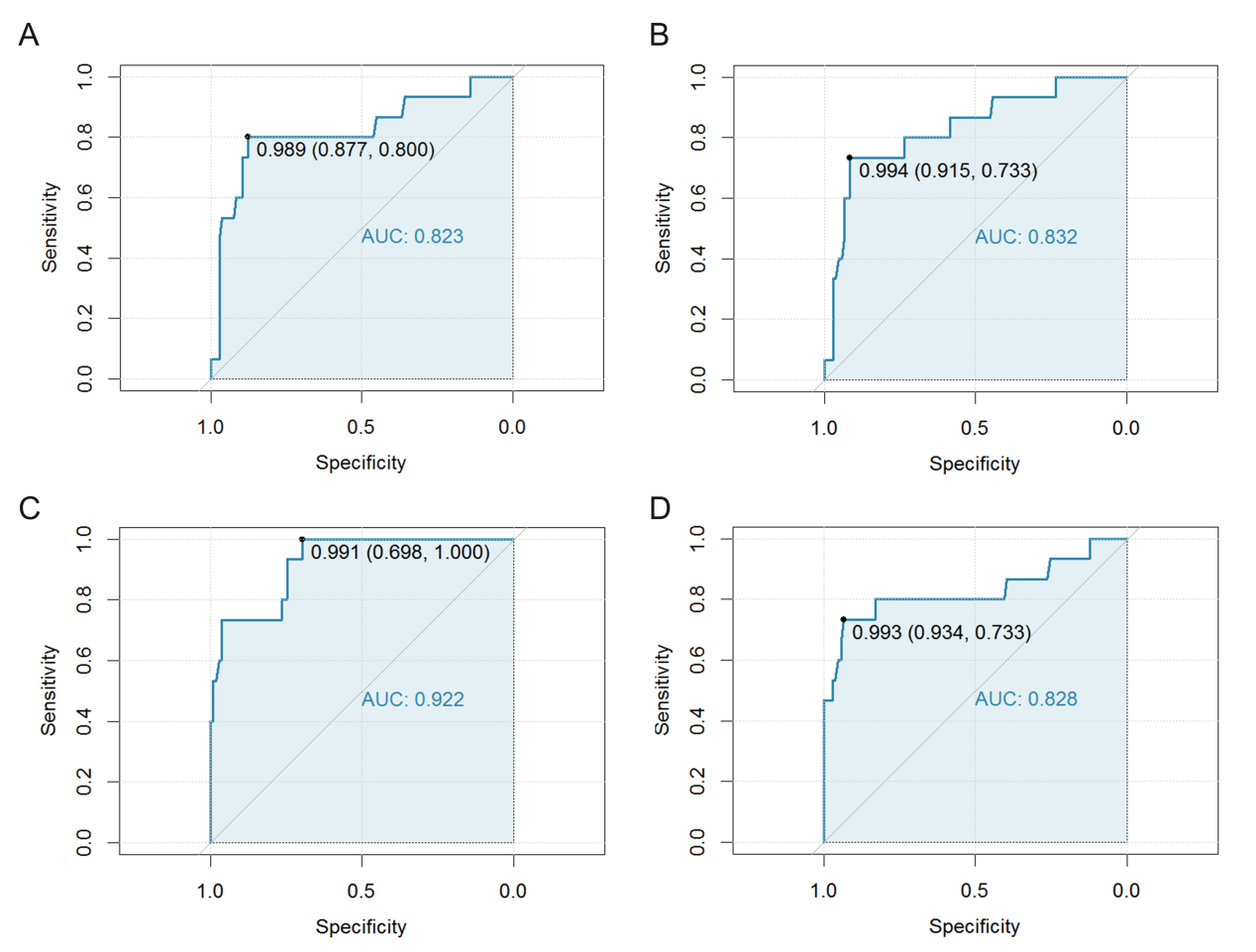


**Figure S3** ROC Curves for Multivariate Analysis‌ of the Severity of Pneumonia

1. Basic information as independent variable. (B) Infection type as independent variable. (C) Symptom as independent variable. (D) Pathogen type as independent variable.
