# Supplementary material for: Post-COVID-19 era pathogen profiles and influencing factors for hospital patients with lower respiratory tract infections in Shenzhen, China
Source: Front Cell Infect Microbiol. 2025 Dec 5;15:1703955. doi: 10.3389/fcimb.2025.1703955 (PMC12714876; doi:10.3389/fcimb.2025.1703955)
Supplement: Supplementary file 6 [file Table6.docx]

**Supplementary Material 6**

**Table S10** ‌Univariate Analysis‌ of LOS

| **Factor** | **Mann-Whitney U** | **Wilcoxon W** | **Effect Size (r)** | ***P*** -value |
| --- | --- | --- | --- | --- |
| **Basic Information** |  |  |  |  |
| Age^a^ |  |  |  | ＜0.001 |
| Gender | 1744.500 | 3284.500 | 0.033 | 0.713 |
| Diabetes Mellitus | 552.500 | 6012.500 | 0.226 | 0.013 |
| Hypertension | 616.500 | 6287.500 | 0.128 | 0.159 |
| **Infection Type** |  |  |  |  |
| Multiple Infections | 884.500 | 2537.500 | 0.445 | ＜0.001 |
| **Symptom** |  |  |  |  |
| Cough | 317.500 | 353.500 | 0.128 | 0.159 |
| Expectoration of Sputum | 1164.500 | 1599.500 | 0.094 | 0.302 |
| Fever | 1256.500 | 4259.500 | 0.215 | 0.018 |
| Shortness of Breath | 973.000 | 4889.000 | 0.254 | 0.005 |
| Rales | 743.500 | 5793.500 | 0.191 | 0.035 |
| Pleural Effusion | 670.500 | 5923.500 | 0.194 | 0.033 |
| Heamoptysis | 417.500 | 6858.500 | 0.033 | 0.718 |
| Chest Pain | 242.500 | 6912.500 | 0.112 | 0.219 |
| **Pathogen Type** |  |  |  |  |
| *S. aureus* | 853.000 | 6209.000 | 0.049 | 0.588 |
| *P. aeruginosa* | 416.000 | 5772.000 | 0.340 | ＜0.001 |
| *H. influenzae* | 694.000 | 6580.000 | 0.006 | 0.946 |
| SARS-CoV-2 | 366.500 | 6582.500 | 0.162 | 0.075 |
| HHV | 302.000 | 6518.000 | 0.217 | 0.017 |
| CMV | 284.000 | 6612.000 | 0.198 | 0.029 |
| *A. baumannii* | 240.500 | 6681.500 | 0.201 | 0.027 |
| *M. pneumonia* | 335.000 | 371.000 | 0.111 | 0.221 |
| IFV-A | 388.000 | 6829.000 | 0.061 | 0.503 |
| *S. pneumoniae* | 335.000 | 7005.000 | 0.011 | 0.905 |

^a^Analysed by Spearman’s rank correlation analysis (rs=0.405).

**Table S11** Multivariate Analysis‌ of LOS

| **Factor** | **EXP(B)** | **CI(95%)** | ***P* -value** | **VIF** |
| --- | --- | --- | --- | --- |
| **Basic Information**^a^ |  |  |  |  |
| Age | 1.012 | 1.005 to 1.018 | ＜0.001 | 1.183 |
| Gender | 1.068 | 0.847 to 1.346 | 0.578 | 1.097 |
| Diabetes Mellitus | 1.418 | 1.004 to 2.003 | 0.048 | 1.226 |
| Hypertension | 1.594 | 1.127 to 2.256 | 0.008 | 1.077 |
| **Infection Type**^b^ |  |  |  |  |
| Multiple Infections | 1.715 | 1.387 to 2.120 | ＜0.001 | 1.145 |
| **Symptom**^c^ |  |  |  |  |
| Fever | 1.199 | 0.947 to 1.519 | 0.132 | 1.194 |
| Shortness of Breath | 1.392 | 1.099 to 1.762 | 0.006 | 1.136 |
| Rales | 1.056 | 0.805 to 1.385 | 0.693 | 1.131 |
| Pleural Effusion | 1.684 | 1.230 to 2.307 | ＜0.001 | 1.243 |
| **Pathogen Type**^d^ |  |  |  |  |
| *P. aeruginosa* | 1.540 | 1.143 to 2.073 | 0.004 | 1.251 |
| SARS-CoV-2 | 1.889 | 1.315 to 2.712 | 0.001 | 1.06 |
| HHV | 1.882 | 1.290 to 2.747 | 0.001 | 1.108 |
| CMV | 1.218 | 0.833 to 1.783 | 0.309 | 1.082 |
| *A. baumannii* | 1.147 | 0.738 to 1.782 | 0.542 | 1.278 |

^a^χ²(4) = 44.114, *P*<0.001.

^b^Adjusted for age, gender, diabetes mellitus and hypertension;χ²(5) = 66.679, *P*<0.001.

^c^Adjusted for age, gender, diabetes mellitus and hypertension;χ²(8) = 70.897, *P*<0.001.

^d^Adjusted for age, gender, diabetes mellitus and hypertension;χ²(9) = 72.882, *P*<0.001.


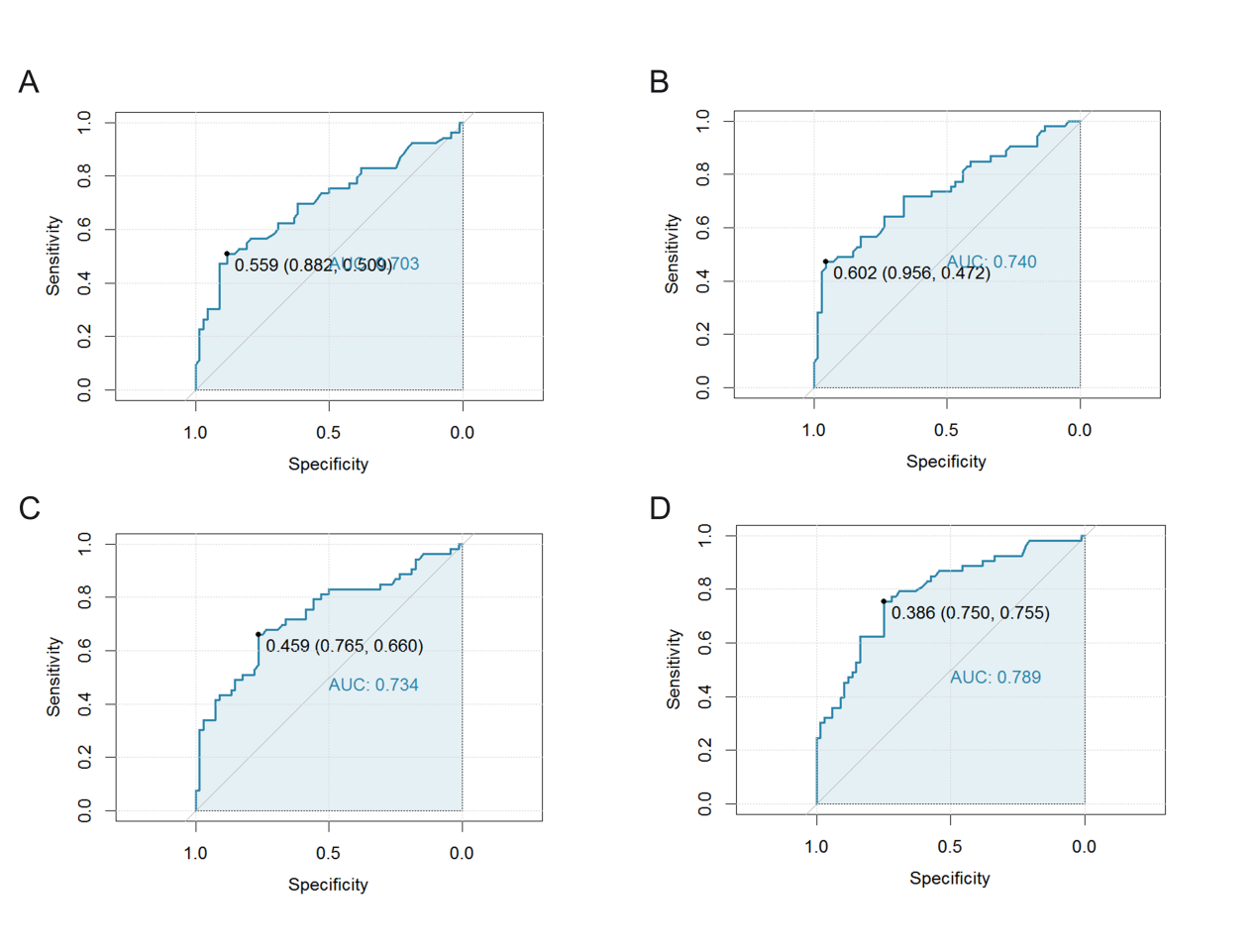


**Figure S4** ROC Curves for Multivariate Analysis‌ of LOS

1. Basic information as independent variable. (B) Infection type as independent variable. (C) Symptom as independent variable. (D) Pathogen type as independent variable.
